# The effect of scenario-based training versus video training on nurse anesthesia students’ basic life support knowledge and skill of cardiopulmonary resuscitation: a quasi-experimental comparative study

**DOI:** 10.1186/s12909-024-05490-3

**Published:** 2024-05-09

**Authors:** Vahid Saidkhani, Masoumeh Albooghobeish, Zahra Rahimpour, Mohammad Hosein Haghighizadeh

**Affiliations:** 1 Department of Anesthesiology, School of Allied Medical Sciences, Ahvaz Jundishapur University of Medical Sciences, Ahvaz, Iran; 2 Department of Biostatistics and Epidemiology, School of Health, Ahvaz Jundishapur University of Medical Sciences, Ahvaz, Iran

**Keywords:** Scenario-based training, Video training, Basic life support, Knowledge, Skill, Cardiopulmonary resuscitation, Anesthesia students, Simulation

## Abstract

**Background:**

Performing CPR (Cardiopulmonary Resuscitation) is an extremely intricate skill whose success depends largely on the level of knowledge and skill of Anesthesiology students. Therefore, this research was conducted to compare the effect of the scenario-based training method as opposed to video training method on nurse anesthesia students’ BLS (Basic Life Support) knowledge and skills.

**Methods:**

This randomized quasi-experimental study involved 45 nurse anesthesia students of Ahvaz Jundishapur University of Medical Sciences, Ahvaz, Iran in 2022–2023. The practical room of the university formed the research environment. The participants were randomly divided into three groups of scenario-based training, video training, and control. Data were collected by a knowledge questionnaire and a BLS skill assessment checklist before and after the intervention.

**Results:**

There was a significant difference between the students’ scores of BLS knowledge and skill before and after the educational intervention in both SG (scenario group) (*p* < 0.001) and VG (video group) (*p* = 0.008) (*p* < 0.001). However, no significant difference was observed in this regard in the CG (control group) (*p* = 0.37) (*p* = 0.16). Also, the mean scores of BLS knowledge and skills in the SG were higher than those in the VG (*p* < 0.001).

**Conclusion:**

Given the beneficial impact of scenario-based education on fostering active participation, critical thinking, utilization of intellectual abilities, and learner creativity, it appears that this approach holds an advantage over video training, particularly when it comes to teaching crucial subjects like Basic Life Support.

## Introduction

Cardiopulmonary arrest refers to the sudden cessation of breathing and blood circulation and resuscitation to restore life to a person who has experienced clinical death [1]. The theoretical concept of CPR (Cardiopulmonary Resuscitation) involves establishing ventilation and blood circulation until spontaneous circulation is restored [2]. In practice, CPR aims to revive the heart and lungs, the two vital organs, in order to restore life. CPR consists of two main components: BLS (Basic Life Support) and ACLS (Advanced Cardiac Life Support) [3]. Cardiovascular disease remains the leading cause of death worldwide [2]. Disciplines such as emergency medicine, cardiology, and anesthesia play a crucial role in managing patients with cardiac arrest. Therefore, it is vital and highly important for individuals in these fields to possess knowledge and familiarity with the principles of cardiopulmonary resuscitation [4]. Cardiopulmonary arrest may happen anywhere, in hospital or outside it, and if CPR is performed adequately and correctly, the patient’s survival rate increases [5]. Performing CPR is an extremely intricate skill whose success depends largely on the level of knowledge and skill of the health care workers [6]. The significance of CPR training for healthcare professionals has been highlighted in a study examining the skills and motivation of residents in fields related to cardiopulmonary resuscitation, such as anesthesia and nursing [7]. It is obvious that efficient and appropriate training can help them in the successful rescue process [8]. Tragic deaths can be prevented through a few hours of theoretical and practical CPR training [7].

Simulation-based training is an effective strategy for teaching CPR and teamwork [9]. Simulation has emerged as one of the most effective learning methodologies in clinical nursing education programs [10]. Unlike conventional teaching methods such as lectures, simulation actively engages learners, promoting a more interactive and participatory learning experience [11]. Simulation has the potential to enhance patient safety by providing a safe and controlled environment for practice [12]. Moreover, the use of simulation can significantly improve the knowledge, skills, and performance of nursing students, enabling them to develop high levels of critical thinking and acquire new professional skills without compromising patient safety [13].

The use of educational videos is the other relatively new method, which is claimed to provide positive results ranging from superficial learning to comprehensive learning. Therefore, it is necessary to assess the effectiveness of new methods such as video education [14]. One of its advantages is the possibility of storing and continuity of information, ease of use, and cost-effectiveness. Also, video speeds up learning and recall and strengthens long-term memory [15]. The findings of Ahn et al. showed that repeated viewing of CPR videos through mobile phones increased retention of the CPR skills of the studied group [16]. Each of the mentioned educational methods have different effects on the trainees, and it is important that right decisions be made in order to avoid possible problems and costs and to put patient safety on priority of care in the health and treatment system. However, the literature lacks sufficient studies regarding the use of an effective educational method to improve the performance and ability of nurse anesthesia students in terms of performing BLS on the injured. Therefore, the present study aims to compare the effect of scenario-based training versus video training on nurse anesthesia students’ BLS knowledge and skills. Finding the preferred teaching method and providing suggestions to be useful for professors of medical sciences are other objectives of this study.

## Method

### Type of study and setting

The research design employed in this study was a quasi-experimental pre-test-post-test intervention design. The study was conducted at Jundishapur University of Medical Sciences in Ahvaz, Iran. Participants consisted of second and third-year Bachelor of anesthesia students. According to the obtained statistics, a total of 21 s-year students and 24 third-year students were studying in the university.

### Sample size and sampling

All students studying in the second and third years were assessed based on the entry criteria. These criteria included being a second- or third-year anesthesia student, providing consent to participate, and not having completed a basic cardiopulmonary resuscitation (CPR) course within the last few months. Additionally, students who did not complete the questionnaires, were absent or did not attend the training sessions, and those who opted out of continued participation were excluded from the study. The sample size for this study was determined based on a previous study [17] and utilizing the sample size formula outlined below.

$$n = \frac{{{{\left( {{Z_{1 - \frac{\alpha }{2}}} + {Z_{1 - \beta }}} \right)}^2}\left( {S_1^2 + S_2^2} \right)}}{{{{\left( d \right)}^2}}} = \frac{{{{\left( {1.96 + 1.28} \right)}^2}\left( {{{1.16}^2} + {{1.27}^2}} \right)}}{{{{\left( {1.7} \right)}^2}}} = 12\,\,\,\,\,\,\,\,\,\,\,\,\,12 \times 3\, = \,36$$

In the final analysis, a total of 45 students were included in the study, accounting for a 10% dropout rate. These participants were then randomly assigned to three groups: two intervention groups (scenario-based training group and video training group) and a control group. Random allocation was achieved using a table of random numbers, wherein each student was assigned a number. Students with numbers 1 to 3 were allocated to the scenario group, those with numbers 4 to 6 were assigned to the video group, and students with numbers 7 to 9 were placed in the control group. Randomization was solely applied in the distribution of samples between the intervention and control groups. Consequently, this research project, like many others in the field of medical education, was conducted in a semi-experimental manner. As a result, 15 individuals were allocated to each group. The students were blinded to their group assignment, meaning they were unaware of whether they were placed in the scenario-based training group, the video training group, or the control group. Additionally, the individual selected by the research team as an examiner to evaluate the resuscitation skills was unaware of the specific training method assigned to each group. This study was conducted between 2022 and 2023. The sampling process began on November 6 and continued for a duration of one week.

### Data collection tools

The data collection tool included three sections: demographic information questionnaire, cardiopulmonary resuscitation knowledge assessment questionnaire and cardiopulmonary resuscitation skill assessment checklist.

#### 1- Demographic information questionnaire

The questionnaire encompassed inquiries regarding age, gender, marital status, academic semester, and prior participation in similar training courses.

#### 2- Cardiopulmonary resuscitation knowledge Assessment Questionnaire

For the preparation of the resuscitation knowledge questionnaire, references such as basic pre-hospital medical emergencies and an introduction to pre-hospital emergency management in the scene of rescue operations and the 2021 guidelines of the European Resuscitation Council were utilized [18]. These sources provided the necessary information and guidance to develop the questionnaire. The knowledge questionnaire consists of 15 four-choice questions, in which correct answers are scored one. The total score ranges from 0 to 15.

**3- Cardiopulmonary Resuscitation Skill Assessment Checklist**: This checklist contains 13 items that are scored based on a 5-point Likert scale (very poor: 1, poor: 2, average: 3, good: 4, very good: 5). The maximum score in this section is 65.

To create the resuscitation skill checklist, various sources were consulted, including references such as pre-hospital primary medical emergencies, an introduction to pre-hospital emergency management at the scene of rescue operations and the 2021 guidelines of the European Resuscitation Council [18]. Additionally, Chegeni et al.‘s study was also utilized as a valuable resource [19].

A panel of experts was used to validate the data collection tool. To this aim, the opinions of ten faculty members who were experts in face and content validity were sought and the required amendments were made. To determine the reliability of the knowledge questionnaire, the test-retest method was used at an interval of ten days, and the obtained Pearson correlation coefficient was 0.87. Internal consistency method was used for the skill checklist, and the obtained Cronbach’s alpha coefficient was 0.74.

CVR: The content validity ratio was calculated using the Lavshe table, resulting in a value of 0.62. It was found that all the items in both the knowledge questionnaire and skill checklist had values higher than 0.62, indicating their necessity and confirming their content validity [20].

CVI: In this study, the content validity index was used to assess the relevance and necessity of the items in the knowledge questionnaire and skill checklist. A CVI score higher than 0.79 was considered suitable, between 0.70 and 0.79 was deemed questionable and in need of correction and revision, and a score less than 0.70 was considered unacceptable and resulted in the removal of the respective items. It was found that all the items in both the knowledge questionnaire and skill checklist had a CVI higher than 0.7, indicating their appropriateness in terms of relevance and necessity [20].

### Data collection

This study was conducted following the acquisition of necessary permits from the Ethics Committee of Jundishapur University of Medical Sciences, Ahvaz, and relevant authorities. The researcher visited the study environment, explained the research objectives to the participants, and obtained written informed consent from them. Subsequently, eligible participants were randomly assigned to three groups: scenario-based training (15 participants), video training group (15 participants), and control group (15 participants). Prior to implementing the intervention, demographic information questionnaires and cardiopulmonary resuscitation knowledge assessments were completed by all participants across the three groups. Additionally, to assess the participants’ cardiopulmonary resuscitation skills before the intervention, each participant underwent individual practice sessions, and their performance was evaluated using a designated checklist. To minimize potential biases, an examiner was selected by the research team to evaluate the participants’ resuscitation skills. The examiner was blinded to the intervention groups and had received training from the researcher on utilizing the checklists. The examiner’s scientific and practical competence was validated by the research team. Subsequently, participants in the first intervention group received scenario-based training, while those in the second intervention group received video training.

In the intervention group of scenario-based education, a two-hour session was conducted wherein theoretical material was presented using a lecture method and PowerPoint slides. Following this, students were divided into several groups, with each group assigned predetermined scenarios corresponding to the educational topic covered in the session. Within their respective groups, students were tasked with identifying the most correct and appropriate scientific and practical actions based on the information acquired during the training session. They collaborated to integrate their answers while utilizing a mannequin to simulate real-life scenarios. Subsequently, one member from each group presented the solutions and fundamental actions related to their assigned scenario to the audience for discussion. Throughout the session, the coach played a pivotal role in monitoring the execution of first aid and resuscitation techniques, as well as providing essential feedback regarding the quality of care delivered by the students.

In the video training intervention group, a two-hour session was conducted with a similar format to the scenario-based education group. The session began with the presentation of theoretical material through lectures and PowerPoint slides, similar to the previous group. Following this, an educational video on basic cardiopulmonary resuscitation, selected by the researcher to align with the session’s content, was shown to the participants.

The educational content presented in both groups included the following: defining and introducing the history of basic cardiopulmonary resuscitation, explaining the importance and functioning of vital systems such as the central nervous system, blood circulation, and breathing, defining and introducing the types of death, symptoms of cardiorespiratory arrest, and cerebral arrest. Additionally, the content covered examining the importance and methods to maintain the safety of oneself and the injured person at the scene of the accident, checking the alertness, pulse, and breathing of the injured person, opening the airway, establishing ventilation, establishing blood circulation and performing extracardiac massage. Moreover, it included introducing the side effects of extracardiac massage and methods for preventing these complications, as well as introducing various resuscitation cases.

The main researcher, a senior student of intelligence education, served as the trainer and led all training activities. The educational materials used in this intervention were derived from basic pre-hospital medical emergency textbooks and provided an introduction to pre-hospital emergency management at the scene of rescue operations.

It should be noted that no educational intervention was performed in the control group. Two weeks after the intervention, the knowledge questionnaire was completed again for all three groups. Additionally, the checklist was individually filled out by the examiner for each participant. Subsequently, the data were collected and subjected to final analysis.

### Data analysis

Data were reported using descriptive statistics. Kolmogorov-Smirnov test has shown the normality of the data. Chi-square test was used to compare qualitative results. Data were analyzed using paired t-test (for pre- and post-test), analysis of covariance, and one-way analysis of variance in SPSS version 20. In all stages of the research, the significance level was considered less than 0.05.

## Results

A total of 45 students participated in the present study. The mean age of the participants was 21.5 ± 3.42 years (minimum 19 and maximum 43 years). In the scenario, video and control groups, the mean age was 20.8 ± 0.99, 22.8 ± 5.68, and 21.0 ± 1.03 years, respectively. One-way analysis of variance was used to compare the mean age in three groups, and the difference was not significant (*p* = 0.23). The three groups were similar in terms of gender, marital status, and academic year (Table 1).

**Table 1:** Demographic characteristics and comparison between the three groups before intervention

Variable	Scenario group *N* = 15	Video group *N* = 15	Control group *N* = 15	*P*-value^*^
**Gender; n (%)**				1.00
Female	10 (66.7)	10 (66.7)	10 (66.7)	
Male	5 (33.3)	5 (33.3)	5 (33.3)	
**Marriage status; n (%)**				0.36
Single	15 (100)	14 (93.3)	15 (100.0)	
Married	0.0 (0.0)	1 (6.7)	0.0 (0.0)	
**Academic year; n (%)**				1.00
Second -year	7 (46.7)	7 (46.7)	7 (46.7)	
Third-year	8 (53.3)	8 (53.3)	8 (53.3)	

* Chi-square test

Paired t-test was used to compare the mean knowledge scores of pre-test and post-test in three groups. The results showed that there was a statistically significant difference between the mean knowledge scores of the pre-test and post-test in the SG (Scenario Group) (*p* < 0.001) and VG (Video Group) (*p* = 0.008), with a significant increased being observed in the mean knowledge scores obtained at the post-test. However, this difference was not statistically significant in the CG (control group) (*p* = 0.37). One-way analysis of variance was used to compare the mean knowledge scores of the pre-test and post-test between the three groups. The results showed that there was no statistically significant difference between the mean knowledge scores of the pre-test in the three investigated groups (*p* = 0.29), but a significant difference was observed between the mean knowledge scores of the post-test in the three groups (*p* < 0.001), with the mean knowledge scores in the SG being higher compared with the VG and CG (Table 2).

**Table 2:** Comparing BLS knowledge scores before and after the training intervention in the three groups

	Scenario	Video	Control	*P*-value*
	Mean (Standard deviation)	Mean (Standard deviation)	Mean (Standard deviation)	
Pretest	5.73 (2.43)	6.73 (1.53)	5.60 (2.29)	0.29
Posttest	11.06 (2.57)	8.66 (1.63)	6.00 (1.55)	<0.001
P-value**	<0.001	0.008	0.37	
	t = 5.42	t = 3.11	t = 0.92	
P-value***<0.001				

*One-way ANOVA

**Paired Sample T-test

***ANCOVA

The results also showed that there was a significant difference between the mean skill scores of pre-test and post-test in the SG (*p* < 0.001) and VG (*p* < 0.001), with a significant increase being observed in the mean skill scores of the post-test. However, this difference was not significant in the CG (*p* = 0.16). There was no statistically significant difference between the mean skill scores of the pre-test in the three investigated groups (*p* = 0.89). However, a significant difference was observed between the mean skill scores of the post-test in the three groups (*p* < 0.001), with the mean skill scores in the SG being higher than that in the VG and CG (Table 3).

**Table 3:** Comparison of BLS skill scores, before and after the training intervention in the three groups

	Scenario	Video	Control	*P*-value*
	Mean (Standard deviation)	Mean (Standard deviation)	Mean (Standard deviation)	
Pretest	20.53 (7.56)	22.06 (9.59)	21.33 (8.74)	0.89
Posttest	42.86 (6.47)	34.00 (8.54)	19.73 (6.45)	<0.001
P-value**	<0.001	<0.001	0.16	
	t = 12.38	t = 6.47	t=-1.48	
P-value***<0.001				
*One-way ANOVA **Paired Sample T-test *** ANCOVA				

The ANCOVA (analysis of covariance) was used to examine the combined effect of time and group, while also controlling for the potential influence of pre-test knowledge and skill scores. the results indicated a statistically significant difference between the two case groups in terms of their mean knowledge and skill scores at the post-test (*p* < 0.001). This means that the intervention had a noticeable impact on the participants’ knowledge and skill levels. Additionally, the effect size was calculated to provide an estimate of the practical significance of the intervention. For knowledge, the effect size was 0.54, indicating a moderate effect. For skill, the effect size was 0.79, suggesting a larger effect. These effect sizes indicate the magnitude of the difference between the groups and provide insights into the practical significance of the intervention. Overall, these findings suggest that the intervention was effective in improving participants’ knowledge and skill levels, with a notable impact observed in both areas.

## Discussion

In the present study, there was a significant difference between the students’ scores of BLS knowledge and skill, before and after the educational intervention based on the scenario and video methods. However, this difference was not significant in the CG, so the training interventions in this study were effective in increasing the students’ BLS knowledge and skills. Laco et al. showed that BLS and urgent care clinic scores of the participants in their study increased after simulation training [21]. Also, Toubasi et al. found that BLS simulation training sessions were associated with a significant improvement in the skills of Jordanian nurses. They also maintained that a BLS training session is highly recommended for nurses to ensure their preparation in real CPR scenarios [22]. In Boada et al.‘s study, the use of a game called LISSA (life support simulation activities) used as a teaching method led to higher scores in acquisition and retention of skills among nursing students trained by LISSA compared to the group that was trained by the traditional method [23]. The results of another study by Ackermann et al. indicate that the use of educational videos leads to enhanced awareness and skills of the participants in terms of CPR [24].

With the unprecedented advancements in science and technology in the last few years, the use of electronic content and educational videos provides an opportunity for the learners to manage their learning according to their personal interests, preferences, and needs. They can also repeat the learning experience as many times as they wish, and by changing the speed at which the video is played they can facilitate their learning and better acquire the details [25]. In the studies cited above, various simulation methods were used for clinical training, which ultimately led to the improvement and promotion of clinical efficiency. Of course, measuring the effectiveness of each educational method alone or in combination with each other in clinical learning requires further research. In the present study, two simulation methods were compared, and a significant difference was observed after both educational interventions. However, the mean scores of knowledge and BLS skills in the SG were significantly higher compared with the VG. This indicates the greater effectiveness of the scenario method which involves the active participation of learners and the use of interactive discussions in this educational method. As Haugland et al. showed in their study, scenario-based simulation is a useful approach to prepare nursing students to be more aware of difficult situations and how to handle them. According to their results, with active participation, learners remember the materials better, and when teaching is based on simulation and theoretical principles, learning is significantly enhanced [26]. Falahinia et al. compared practical training with video training as two educational methods and found that both methods are equally effective, but using training videos can offer advantages such as easy use, cost-effectiveness, repeatability of the content, and no need for student presence in class [25]. In Erenel et al., it was shown that although the scenario-based simulation method was used by educators to increase students’ preparation for practice, it led to a decrease in clinical satisfaction, decreased the students’ stress, and had no effect on the students’ self-confidence level [27]. The findings of Baek et al.‘s study showed that this educational method helps to develop nursing competence in nursing students without experience in nursing internship, and the authors recommended inclusion of developing scenario learning in the nursing curriculum to promote initial adaptation to clinical environments [28]. Based on the results of these studies, it can be concluded that given the nurse anesthesia students’ dire need for preparation and effective performance of CPR, a novel and efficient teaching method should be adopted for teaching these students. Quality and effective training methods, such as the scenario-based method, will lead to the learners’ enhanced level of knowledge, self-confidence, and courage during resuscitation operations, giving them the ability to act independently and more successfully in saving the lives of thousands of patients. Of course, it should be noted that in case it is not possible to use more effective methods such as scenario-based training, video training seems to be the best alternative.

This study has several strengths worth mentioning. Firstly, it stands out for its inclusion of a control group, setting it apart from other similar studies. Additionally, unlike previous research that primarily compared simulation methods with traditional lecture-based teaching, this study specifically compares two simulation-based teaching methods. This approach provides a more comprehensive understanding of the effectiveness of different instructional techniques. Lastly, while many studies solely focus on the impact of simulation-based teaching methods, this study also investigates their effect on student learning outcomes.

### Limitations of the study

Due to our limited educational facilities and space, as well as the less active participation of students in research, it was not possible to design and implement stronger crisis scenarios. In this study, only the components of knowledge and skills were examined, and factors such as the effect of educational methods on the level of satisfaction, stress, and self-confidence of students were not examined, which can be addressed in future studies. Also, a larger sample size can be used to achieve more accurate results with greater generalizability.

## Conclusion

According to the results of the present study, the increase in the nurse anesthesia students’ level of scientific knowledge and practical skills of BLS after intervention was higher in the SG compared with the VG. Therefore, it can be concluded that the educational method based on the scenario is more efficient and effective than the other teaching method. In addition, it should be noted that due to the scarcity of similar research, future studies are recommended to examine the effect of new educational methods and compare them with each other, especially the effect of using different simulation-based teaching methods for teaching basic and advanced CPR and other emergency situations.

## Data Availability

All data generated or analyzed during this study are included in this published article.
